# Phospholipogenic Pharmaceuticals Are Associated with a Higher Incidence of Histological Findings than Nonphospholipogenic Pharmaceuticals in Preclinical Toxicology Studies

**DOI:** 10.1155/2012/308594

**Published:** 2012-06-14

**Authors:** Linda R. Barone, Scott Boyer, James R. Damewood, James Fikes, Paul J. Ciaccio

**Affiliations:** ^1^Biologics Research, Janssen Research and Development, LLC, 145 King of Prussia Road, Radnor, PA 19087, USA; ^2^Safety Assessment, AstraZeneca Pharmaceuticals, Pepparedsleden 1, 431 83 Mölndal, Sweden; ^3^Haskell Global Centers for Health and Environmental Sciences, DuPont Central Research and Development, Newark, DE 19711, USA; ^4^Safety Assessment, AstraZeneca Pharmaceuticals, 35 Gatehouse Drive, Waltham, MA 02451, USA

## Abstract

While phospholipidosis is thought to be an adaptive response to chemical challenge, many phospholipogenic compounds are known to display adverse effects in preclinical species and humans. To investigate the link between phospholipogenic administration and incidence of preclinical histological signals, an internal AstraZeneca *in vivo *toxicology report database was searched to identify phospholipogenic and nonphospholipogenic compounds. The datasets assembled comprised 46 phospholipogenic and 62 nonphospholipogenic compounds. The phospholipogenic potential of these compounds was confirmed by a pathologist's interpretation and was supported by well-validated *in silico *and *in vitro *models. The phospholipogenic dataset contained 37 bases, 4 neutral compounds, 3 zwitterions, and 1 acid, whereas the nonphospholipogenic dataset contained 9 bases, 34 neutrals, 1 zwitterion, and 18 acids. Histologic findings were tracked for adrenal gland; bone marrow; kidney; liver; lung; lymph node; spleen; thymus; and reproductive organs. On average, plasma exposures were higher in animals dosed with the nonphospholipogenics. Phospholipogenics yielded proportionally more histologic changes (exclusive of phospholipidosis itself) in all organs. Statistically significant higher frequencies of liver necrosis, alveolitis/pneumonitis, as well as lymphocytolysis in the thymus, lymph nodes, and spleen occurred in response to phospholipogenics compared to that for nonphospholipogenics.

## 1. Introduction

Drug-induced phospholipidosis (PLD) is a lipid storage disorder characterized by the intralysosomal accumulation of polar lipids, membranous lamellar inclusions, and drug. The cytological features of PLD mimic inherited lipidoses [1]. Common primary tissue targets are alveolar macrophages, hepatic Kupffer cells, bile duct epithelia, and peripheral blood mononuclear cells, although numerous other cellular targets have been reported. Many marketed and experimental drugs have been reported to cause PLD preclinically [2]. Most have basic and hydrophobic physicochemical properties (amphiphilic cations) that enable binding with phospholipids, crossing of biological membranes, and lysomotropism (partitioning of drug into lysosomes once it is protonated at acidic intralysosomal pH) [3]. Lysomotropic properties and drug accumulation potential are underscored by studies employing lysosomal modulators which have a demonstrated ability to abolish the pH gradient from cytoplasm to lysosome, preclude accumulation of drug in the lysosome (depot effect), and/or rescue cells *in vitro* [4–6].

There is a consensus view that PLD itself is largely an adaptive rather than an adverse response to xenobiotics as a number of *ex vivo* studies have failed to consistently reveal specific functional detriment in responding phospholipidotic tissues [2, 7–9]. Yet there is suggestive evidence that phospholipogenic compounds are associated with concurrent toxicities preclinically and clinically. These toxicities include liver necrosis (amiodarone), motor neuropathies (imipramine, amitriptyline), proximal tubule kidney injury (gentamicin), pulmonary alveolitis that can progresses to fibrosis (amiodarone), pneumonitis (fluoxetine), peripheral neuropathies (suramin, amiodarone, chloroquine, perhexiline), and corneal opacity (chloroquine, amiodarone) [10–15]. Despite this potential association, to date, there have been no comprehensive published reviews of pharmaceutical industry data that enumerate these risks or that probe the statistical connection between phospholipogenic compounds and concurrent toxicities. Understanding whether such a connection exists would help define the relative importance of thorough pathological characterization of phospholipidosis and concurrent toxicities in early drug discovery and assist in reducing attrition in drug development.

To investigate the link between phospholipogenic compound administration and the incidence of preclinical histological signals, phospholipogenic and nonphospholipogenic compounds were identified in an internal AstraZeneca *in vivo* toxicology report database and their histological results annotated. We herein report on a robust, unbiased analysis revealing that *in vivo* phospholipogenic compounds are statistically more likely to demonstrate non-PLD histologic changes in all organs investigated. This observation is even more compelling when one considers that, in these studies, the nominal dose (or exposures) for nonphospholipogenic compounds typically exceeded that for phospholipogenic compounds.

## 2. Methods

### 2.1. Compound Identification

An internal AstraZeneca database containing repeat-dose *in vivo* toxicology reports was searched to identify phospholipogenic compounds using the common PLD indicator terms phospholipidosis, histiocytosis, foamy macrophages, lamellar lysosomes, and myeloid bodies. To identify reports on nonphospholipogenic compounds, the database was searched for the absence of these PLD indicator terms. Species, study duration, histological findings, nominal doses, and plasma exposures were the key data extracted. We focused on nine responding organs: adrenal gland; bone marrow; kidney; liver; lung; lymph node; reproductive organs; spleen; thymus. (While we have observed nervous and muscle tissue phospholipidosis in our studies, reports were not archived in time, or search terms were inadequate for the purpose of performing an unbiased analysis.)

Reports were individually read to verify the presence or absence of indicator terms, and both PLD and all non-PLD histologic findings were recorded and annotated. Although PLD often presents as vacuolation by light microscopy, due to its common occurrence, by itself, this finding was not considered sufficient for PLD classification. One or more of the following was considered an additional confirming measure for membership in the phospholipogenic dataset: a positive result in an *in vitro* PLD assay [16], a positive prediction from our in-house *in silico* PLD model, a pathologist interpretation/description of PLD in the report (PLD had to be considered by the pathologist a familiar and salient finding and not that of background), or an electron microscopic finding of PLD, the latter being the gold standard for detecting lysosomal phospholipid-laden myeloid inclusions indicative of PLD. One or more of the following was considered an additional confirming measure of a compound having a nonphospholipogenic status: a negative result in an *in vitro* PLD assay or a negative result in our *in silico *PLD predictive model.

### 2.2. Pharmacokinetic Properties

Nominal dose (*μ*mol/kg), AUC_*μ*m·hr_ and C_max_ (*μ*M) plasma exposure data for the two compound classes were extracted from the study reports if available (Table 1). The majority of the study reports had three dose groups. If a study had four dose groups, the second lowest dose was designated as the low dose. AUC exposure information for at least two doses was available for 77% of the phospholipogenic reports and 86% of the negative's reports; C_max_ exposure information for at least 2 doses was available for 73% of the phospholipogenic compounds and 79% of the nonphospholipogenic compounds. Exposures were not adjusted for plasma protein binding as complete measurements were not available for a majority of the studies.

### 2.3. Statistical Analysis

 Statistically significant differences between the two datasets for organ histological findings were assessed using Chi Square analysis at a 95% confidence level. Statistically significant differences are indicated by Chi Square values greater than 3.84 in Table 2. 

## 3. Results

### 3.1. Source Data for Phospholipogenic and Nonphopholipogenic Compounds

*In vivo* data extracted from an internal *in vivo* toxicology report archive resulted in compilation of forty-six phospholipogenic compounds and sixty-two nonphospholipogenic compounds. To obtain the forty-six phospholipogenic compound set, fifty-three compounds initially identified as phospholipogenics based on verbal descriptors in the study reports were tested in our *in vitro* PLD assay [16], found to be positive, and selected for further analysis. The majority of these compounds exhibited *in vitro* EC_50_ values less than 50 *μ*M (mean 44 *μ*M, sd 66; ranging from 2.4 to 273 *μ*M). For comparison, the phospholipogenics amiodarone, chloroquine, and citalopram yield 4.8, 12, and 38 *μ*M EC_50_ values in this assay, demonstrating that many positives selected for analysis were intrinsically weaker in their phospholipidosis inducing potential than these standard reference drugs.

### 3.2. Physiochemical Properties Analyses

The 107 compounds in the phospholipogenic and nonphospholipogenic datasets were structurally diverse. Clustering based on 2-dimensional molecular fingerprints resulted in seventy-five sets of compounds, with the largest cluster containing eight compounds. Physical and computed molecular properties also revealed this diversity, with molecular weights (MW) ranging from 139 to 696 (mean 434, sd 115), polar surface area (PSA) between 20.5 and 194.9 (mean 85.9, sd 33.4), and ClogP between −2.53 and 8.95 (mean 3.0, sd 2.0). The number of hydrogen bond donors ranged from 0 to 4 (mean 1.7, sd 1.2) and hydrogen bond acceptors from 2 to 14 (mean 6.9, sd 2.4). At pH 7, the phospholipogenic compounds were predicted to contain 37 bases, 4 neutrals, 3 zwitterions, and 1 acid, while the nonphospholipogenic compounds contain 9 bases, 34 neutrals, 1 zwitterion, and 18 acids. This is consistent with the generalization that many phospholipogenic compounds are amphiphilic cations. The MW for the phospholipogenic compounds ranged between 256 and 645 (mean 476, sd 85) and 139 and 695 (mean 404, sd 124) for the negative compounds. ClogP for the phospholipogenic compounds range between 0.67 and 8.95 (mean 3.8, sd 1.6) and for the negative compounds between −2.53 and 7.46 (mean 2.4, sd 2.0). PSA for the phospholipogenics ranged between 30.6 and 138.1 (mean 76.4, sd 25), while, for the nonphospholipogenic compounds it ranges between 20.5 and 194.9 (mean 92.8, sd 37.2). The number of hydrogen bond donors ranges between 0 and 4 (mean 1.5, sd 1.1) and the number of hydrogen bond acceptors between 3 and 12 (mean 6.9, sd 1.8) for the phospholipogenic compounds and between 0 and 4 (mean 1.8, sd 1.2) and 2 and 14 (mean 6.9, sd 2.7), respectively, for the negatives. Aside from the obvious difference in the ionization characteristics of the two sets, these simple physical chemical parameters alone do not appear to effectively distinguish between phospholipogenic and nonphospholipogenic compounds.

### 3.3. Species, Study Durations, and Plasma Exposures

**Figure 1:**
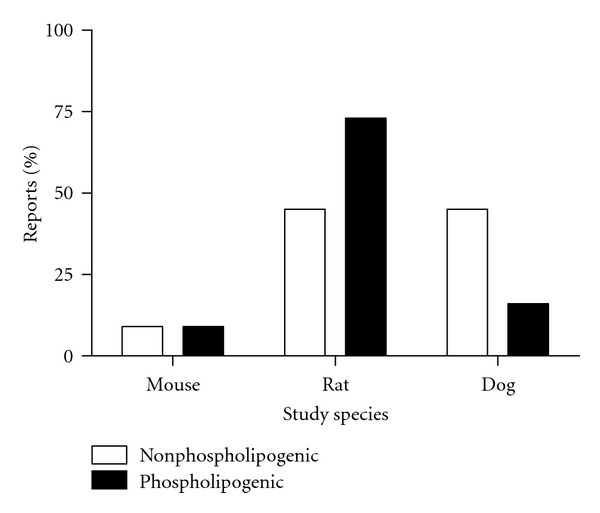
Percentage of study reports by species for both phospholipogenic and nonphospholipogenic datasets. Where there were multiple study reports for a compound tested in the same species, reports were counted as a single “report” to minimize weighting bias. There were 29% (of the total) (or 1.6-fold) more rat study “reports” available in the phospholipogenic dataset than the negatives dataset, whereas there were 28% (of the total) (or 2.8-fold) more dog study “reports” available in the negative dataset than the phospholipogenic dataset. Phospholipidosis potential (descriptors and pathologists interpretation) identified in study reports for all compounds was also confirmed *in silico* or *in vitro*.

**Figure 2:**
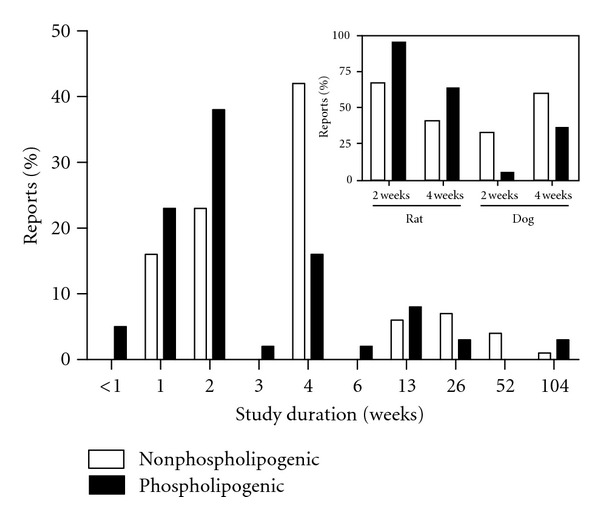
Percentage of study reports for both nonphospholipogenic and phospholipogenic datasets by study duration. Where there were multiple study reports for a compound tested in the same species, reports were counted as a single “report” to minimize weighting bias. There were 15% (of the total) (or 1.7-fold) more 2-week “reports” available for the phospholpogenic dataset than for the negatives; there were 26% (of total) (or 1.7-fold) more 4-week “reports” available for the negative dataset than for the phospholpogenics. The larger figure reflects data pooled from the three species. The *inset *shows the breakdown of two-week and four-week studies by dog and rat species.

Mouse, rat, and dog are the three species represented in the compound datasets. Study durations ranged from five days to two years, with the majority of repeat dose studies having been conducted for 1 to 4 weeks duration.  Figure 1 shows the percentage of study reports by species.  Figure 2 shows the percentage of study reports by study duration. There were on average 4.8 (4.6 sd) and 3.2 (2.3 sd) reports per compound in the phospholipogenic and nonphospholipogenic datasets, respectively. Where there were multiple study reports for a compound tested in the same species, reports were counted as a single “report” to minimize weighting bias. There were some notable differences in the distribution of study species and duration for both compound classes. There were 29% (of the total) (or 1.6-fold) more rat study “reports” available in the phospholipogenic dataset than the negatives dataset, whereas there were 28% (of the total) (or 2.8-fold) more dog study “reports” available in the negative dataset than the phospholipogenic dataset. The percentage of mouse reports was equivalent for both compound classes. The two-week and four-week groupings had the greatest variation in study duration distribution. There were 15% (of the total) (or 1.7-fold) more 2-week “reports” available for the phospholpogenic dataset than for the negatives; there were 26% (of total) (or 1.7-fold) more “reports” available for the negative dataset than for the phospholpogenics. Except for mouse where AUC_*μ*m·hr_ and C_max_ (*μ*M) exposure data were similar between datasets at high nominal doses, nominal dose (*μ*mol/kg), AUC_*μ*m·hr_, and C_max_ (*μ*M) values for the negative compounds were on average higher for the other two species (Table 1). C_max_ exposures from the nonphospholipogenic bin on average well exceeded 10 *μ*M.

#### 3.3.1. Phospholipogenic Compound Incidence of Organ PLD versus Non-PLD Findings

**Figure 3:**
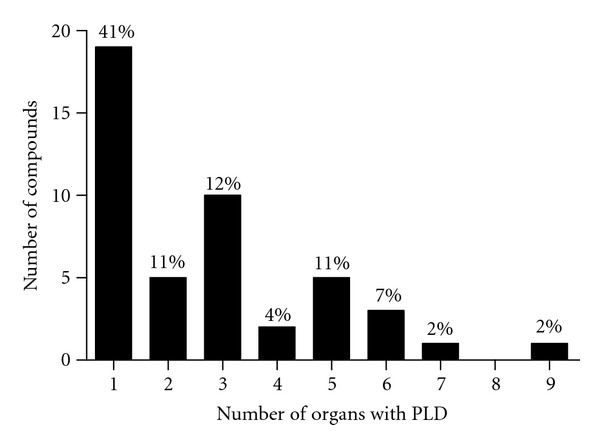
Number of phospholipogenic compounds by incidence of organ phospholipidosis responses. The values above the bars represent proportions of the compounds yielding a phospholipidosis response descriptor (i.e., “phospholipidosis,” “histiocytosis,” “foamy macrophages,” “lamellar lysosomes,” and “myeloid bodies” and where phospholipidosis potential was confirmed by *in silico* or *in vitro* testing). Approximately half of the compounds caused multiorgan PLD (i.e., ≥3 organs) phospholipogenics. The data represent that pooled from three species and all study durations.

**Figure 4:**
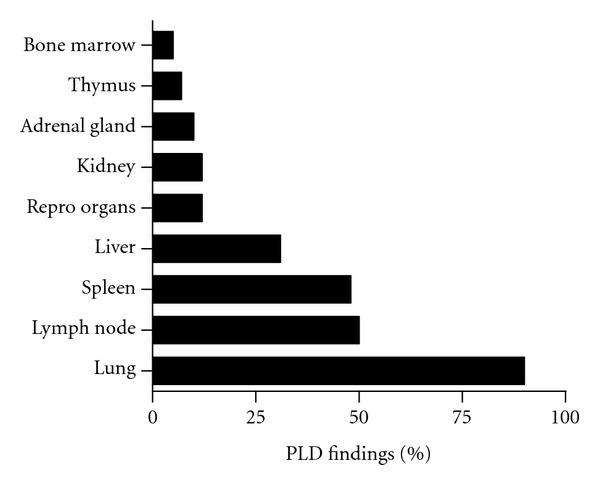
Percent phospholipogenics giving histological findings consistent with phospholipidosis, by organ. In response to phospholipogenic administration, lung and lymph node presented with the greatest number of histological findings that matched phospholipidosis descriptors whereas bone marrow and thymus presented with the least. The data represent that pooled from three species and all study durations.

**Figure 5:**
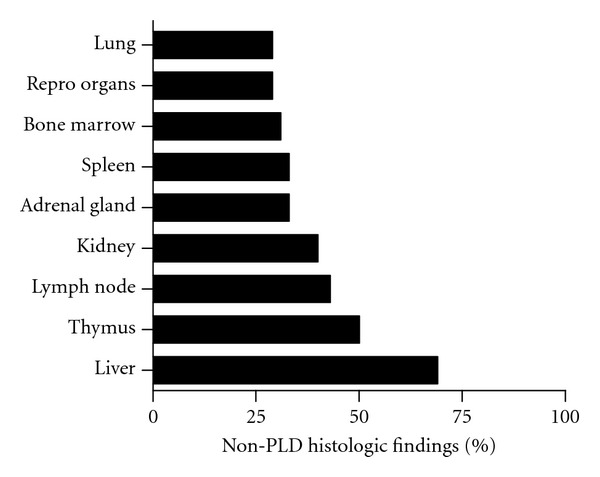
Percent phospholipogenics with nonphospholipogenic histological findings by organ. The data represent that pooled from three species and all study durations.

Compound treatments yielding three or more organ PLD responses were deemed multiorgan phospholipogenic compounds. Approximately 50% of phospholipogenic compounds caused multiorgan PLD (Figure 3). Multiorgan PLD was more likely to occur when predicted percent free compound (free of protein binding) was greater than ~10%, whereas PLD more often occurred in only one to two organs when the predicted percent free was less than 10%. PLD incidence by organ is illustrated in Figure 4. Lung, lymph node, and spleen presented with the highest incidence, bone marrow, and thymus the lowest. The liver PLD incidence was approximately 35%. In contrast, the rank order of non-PLD histologic findings incidence for this dataset differed from that of organ PLD ranking (Figure 5). Liver and thymus exhibited the highest incidences of non-PLD findings, lung, and bone marrow the lowest.

### 3.4. Incidence of Organ Non-PLD Histologic Findings, Comparing Phospholipogenic, and Nonphospholipogenic Datasets

**Figure 6:**
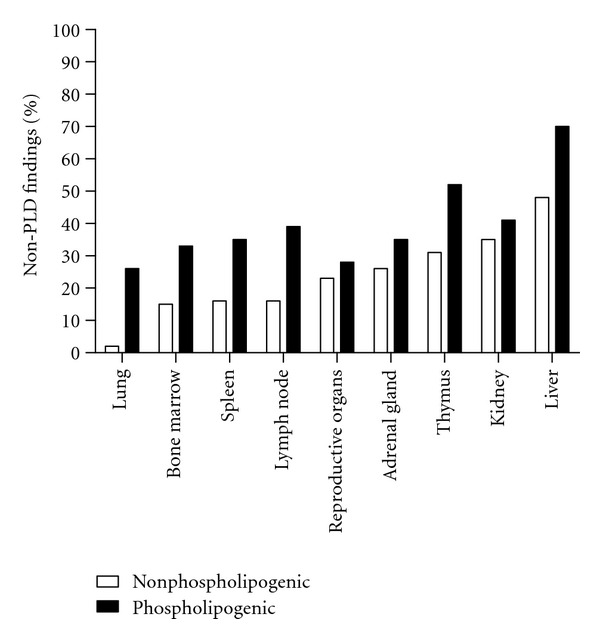
Percent nonphospholipogenic and phospholipogenic compound administrations yielding non-PLD histological findings, by organ. For every organ, on average, phospholipogenic administration resulted in more non-PLD histological findings than did negative compound administration. The data represent that pooled from three species and all study durations.

To ascertain which dataset was associated with more histologic findings, frequencies of non-PLD histologic findings were compared. Phospholipogenic compounds consistently exhibited more non-PLD histologic changes for all organs (Figure 6), and among these the liver exhibited the highest percentage of histologic findings for both datasets. Compared to nonphospholipogenic compounds, phospholipogenic compounds yielded a statistically significant higher percentage of liver necrosis findings (Table 2). For those phospholipogenic compounds in this subset where exposure data were available (i.e., 11 of 22 compounds that caused necrosis), top plasma exposures did not on average exceed those phospholipogenics that did not cause necrosis (data not shown). A statistically significant, higher frequency of lymphocytolysis/lymphoid depletion in lymphoid organs increased apoptosis in spleen, and alveolitis/pneumonitis in lung were observed with this subset of compounds. No differences were apparent for adrenals or kidney. Finally, statistically significant higher levels of “no histologic findings” in the liver, lung, thymus, lymph nodes, and spleen were reported for the nonphospholipogenic compounds.

## 4. Discussion and Conclusions

The purpose of this study was to test whether pharmaceutically relevant phospholipogenic compounds were associated with a higher frequency of histologic changes in repeat dose *in vivo* preclinical toxicology studies than that for nonphospholipogenic compounds. For the comparison, forty-six phospholipogenic compounds were identified; sixty-two negative compounds were identified. Results of the analysis may be summarized as follows. Phospholipogenic administrations were found to yield proportionally more non-PLD histologic changes in general in all organs examined and for select histological findings in liver, lung, thymus, lymph node, and spleen but not for kidney and adrenal glands. Nonphospholipogenic compounds exhibited a higher incidence of “no findings” for several organs. Phospholipogenic compounds exhibited statistically significant increases in lung alveolitis/pneumonitis and in true toxicities relating to liver necrosis. Not surprisingly, phospholipogenic compounds demonstrating “no liver findings” tended to exhibit fewer histological findings across the 9 organs overall (data not shown). Increased incidences of lymphocytolysis and/or lymphoid depletion in three lymphoid tissues (thymus, lymph nodes, and spleen) were also observed in the phospholipogenic dataset. Lymphocytolysis/lymphoid depletion is often attributed to high-dose-mediated stress responses; but they may in some instances represent a true toxicological response. Without other hematologic correlates it is not possible to shed light on this matter, and for this reason labeled these results conservatively simply as excess histological findings. Finally, it also appears from the study duration analysis that two-week study designs and the rat model are typically sufficient to capture phospholipidotic signals *in vivo*. Although concurrent toxicities were not specified, it is of interest that a recent report indicates that a 24 h duration may be sufficient to detect/predict phospholipidosis accurately by employing a small biomarker set in toxicogenomic evaluation [17].

Several additional comparisons were made for the phospholipogenic dataset, particularly regarding physical-chemical properties and toxicokinetic behavior. Consistent with the well-described cationic amphiphilic nature of phospholipogenic compounds, the phospholipogenic dataset was comprised mostly of amphiphilic bases and the negative dataset contained primarily neutrals and acids with only a few bases. Plasma exposures tended to be lower in animals dosed with phospholipogenic compounds. For the dog and mouse, this pharmacokinetic behavior can be explained in part by the selection of lower phospholipogenic compound nominal doses in the study designs but not entirely since relative exposures are still lower if dose-normalized (data not shown). Nominal doses for rat were on average higher than that of the nonphospholipogenic dataset, but plasma exposures in the phospholipogenic dataset were considerably lower. There is an acknowledged general tendency for bases, and particularly lipophilic bases, to have higher volumes of distribution [18], although it has recently been demonstrated that this property alone does not differentiate phospholipogenics from nonphospholipogenics [19]. Finally, it should be noted that not only were the average doses and exposures of the nonphospholipogenic compounds higher, but, as can be seen in Figure 2, the average duration of their studies is somewhat longer as well. All of these factors should bias the number of nonphospholipidosis histological findings profile towards the higher dose, higher exposure, longer study duration group, but, as demonstrated in this study, we see the opposite.

This thorough and systematic analysis of a large preclinical toxicology dataset containing both confirmed phospholipogenics and nonphospholipogenic compounds indicates that phospholipogenic administration is likely to be associated with more and various histological findings, including liver toxicities, than do nonphospholipogenic administrations. The data, while compelling, are correlative and cannot be used to prove causality between phospholipidosis and toxicities. It should also be noted that speculation on functional compromise cannot be made and that histologic findings identified in this exercise were not equal in severity. Findings ranged from relatively benign to severe, and we did not apply risk assessment analysis where it may be found that compounds could proceed normally through the drug development process, taking concurrent toxicities, tissues affected, severity, and safety margins (therapeutic window) into account. Even with these caveats, it is clear that phospholipogenic compounds tend to produce more histological findings and may require additional monitoring and risk assessment, thereby increasing the overall burden on a drug development project.

## Figures and Tables

**Table 1:** Nominal doses and exposures from both the nonphospholipogenic compound (NP) and phospholipogenic compound (P) datasets. Averages and standard deviations (in parentheses) shown. Data from mid-doses are not shown. Average phospholipogenic plasma exposures were lower for all species across all doses than exposures from the negative dataset, but differences were not statistically significant.

Species	Low dose (*μ*mol/kg)	Low C_max_ (*μ*M)	Low AUC (*μ*M·hr)	High dose (*μ*mol/kg)	High C_max_ (*μ*M)	High AUC (*μ*M_·_hr)
Dog (NP)	89 (124)	52 (71)	297 (429)	936 (1266)	253 (334)	2561 (4457)
Dog (P)	47 (67)	9 (13)	102 (177)	467 (614)	27 (20)	335 (345)
Mouse (NP)	323 (182)	15 (16)	52 (60)	2018 (1437)	56 (71)	322 (519)
Mouse (P)	117 (83)	6 (5)	65 (80)	958 (635)	62 (108)	320 (446)
Rat (NP)	162 (356)	25 (51)	224 (671)	1351 (2659)	204 (390)	1377 (2607)
Rat (P)	235 (384)	9 (21)	117 (324)	737 (802)	26 (32)	372 (485)

**Table 2:** Incidence of nonphospholipidosis histological findings (HFs) for liver, kidney, lung, adrenals, and lymphoid tissues for 46 phospholipogenics and 62 nonphospholpogenics. Results are presented as the total number of compounds exhibiting (or not exhibiting—“No findings”) the given histological finding and the percentage of the total number of compounds in that category. ChiSquare values of ≥3.84 conferred statistical significance at *P* < 0.05*. Statistical significance was achieved for select histological findings in liver, lung, thymus, lymph node, and spleen but not kidney and adrenal glands. HF: histological findings.

	Phospholipogenics		Nonphospholipogenics		Chi-Sq*
	Total number	Percent of total compounds	Total number	Percent of total compounds	
Liver					

Necrosis/degeneration	22	48	13	21	8.7*
Hypertrophy	12	26	11	18	1.1
Reduced glycogen vacuolation	8	17	5	8	2.2
No findings	14	30	32	52	4.8*

Kidney					

Necrosis/degeneration	8	17	6	10	1.4
Nephropathy	5	11	5	8	0.25
No findings	27	59	40	65	0.28

Lung					

Pneumonitis	5	11	0	0	7.1*
Oedema	3	7	1	2	1.8
Alveolitis	3	7	0	0	4.2*
No findings	34	74	61	98	14.9*

Adrenal gland					

Hypertophy	5	11	5	8	0.25
Necrosis	4	9	2	3	1.5
No findings	30	65	46	74	1.1

Thymus					

Lymphocytolysis, lymphoid depletion	16	35	6	10	10.3*
Atrophy/involution	13	28	16	26	0.08
No findings	22	48	43	69	5.1

Lymph node					

Lymphocytolysis, lymphoid depletion	12	26	1	2	14.9*
Atrophy	2	4	5	8	0.6
No findings	28	61	52	84	7.3*

Spleen					

Lymphocytolysis, lymphoid depletion	8	17	0	0	11.7*
Altered hematopoiesis	4	9	6	10	0.01
Atrophy	4	9	3	5	0.65
Apoptosis	3	7	0	0	4.2*
No findings	30	65	52	84	5.0*
